# The Role of Social Relationship Quality in Women Diagnosed with Fibromyalgia

**DOI:** 10.1093/geroni/igaf122.3001

**Published:** 2025-12-31

**Authors:** Minju Seong, Hyunjoo An, Inhye Jung, Hyo Jung Lee

**Affiliations:** Yonsei University, Seoul, Republic of Korea; Yonsei University, Seoul, Republic of Korea; Yonsei University, Seoul, Republic of Korea; Yonsei University, Seoul, Republic of Korea

## Abstract

Fibromyalgia is a syndrome characterized by widespread chronic pain, fatigue, sleep disturbance, and depression. Though its severe pain symptoms disrupt individuals’ daily life, the invisible nature of pain and the lack of effective treatment add to patients’ suffering. While the exact cause of onset of this syndrome remains unclear, about 90% of fibromyalgia patients are women and research on its contributing factors such as life stressors is still developing. Thus, this study investigates the relationship quality of older women with family and friends and its association with fibromyalgia diagnosis according to biopsychosocial model. Using data from the Health and Retirement Study (HRS, 2016, 2018, 2019), the study sample consisted of women aged 50 and older who completed the 2019 Health Study Survey and reported information on their fibromyalgia diagnosis (n = 1,900). The relationship quality with family (spouse, children, and relatives) and friends was measured using both positive and negative dimensions. We conducted Chi-square tests and logistic regression models. Findings suggest that both positive (OR=.647, p<.05) and negative (OR=.619, p<.05) spousal relationship scores were linked to a lower likelihood of fibromyalgia diagnosis. However, negative relationship scores with other family members were associated with a higher risk of being diagnosed with fibromyalgia (OR = 1.502, p≤.01). These results further emphasize the need to consider psychosocial factors when dealing with fibromyalgia patients. Family, as the most intimate social network for individuals, may affect the onset of the condition not only in a genetic context, but also through interpersonal interactions.

